# Tetraspanin Assemblies in Virus Infection

**DOI:** 10.3389/fimmu.2018.01140

**Published:** 2018-05-25

**Authors:** Luise Florin, Thorsten Lang

**Affiliations:** ^1^Department of Medical Microbiology and Hygiene, University Medical Center of the Johannes Gutenberg University, Mainz, Germany; ^2^Department of Membrane Biochemistry, Life & Medical Sciences Institute, University of Bonn, Bonn, Germany

**Keywords:** tetraspanin, microdomain, virus, entry, endocytosis, trafficking, budding, receptor

## Abstract

Tetraspanins (Tspans) are a family of four-span transmembrane proteins, known as plasma membrane “master organizers.” They form Tspan-enriched microdomains (TEMs or TERMs) through lateral association with one another and other membrane proteins. If multiple microdomains associate with each other, larger platforms can form. For infection, viruses interact with multiple cell surface components, including receptors, activating proteases, and signaling molecules. It appears that Tspans, such as CD151, CD82, CD81, CD63, CD9, Tspan9, and Tspan7, coordinate these associations by concentrating the interacting partners into Tspan platforms. In addition to mediating viral attachment and entry, these platforms may also be involved in intracellular trafficking of internalized viruses and assist in defining virus assembly and exit sites. In conclusion, Tspans play a role in viral infection at different stages of the virus replication cycle. The present review highlights recently published data on this topic, with a focus on events at the plasma membrane. In light of these findings, we propose a model for how Tspan interactions may organize cofactors for viral infection into distinct molecular platforms.

## Introduction

The contents of the cell are protected from the extracellular surroundings by the plasma membrane: a lipid bilayer densely populated with protein (1, 2). These proteins are specifically distributed throughout the membrane, a phenomenon associated with lipid microdomains, rafts, phases, or clusters. Local enrichments can be explained by spontaneous self-organization driven by thermodynamic principles (3). Conversely, the composition and architecture of membrane proteins is also actively remodeled in order to control specific functions.

Viruses are genetic entities that can form particles of sizes up to 200 nm and require multiple steps to overcome the cell barrier during entry and egress. To gain access into the cell, viruses employ different host receptors, proteases, and signaling molecules. After internalization *via* endocytosis, non-enveloped viruses escape the membranous organelle system in order to deliver viral genetic information into the cytoplasm or nucleus (4–6). Entry of enveloped viruses occurs through fusion of the viral and cellular membrane at the plasma membrane or in intracellular compartments (7). Each viral entry mechanism involves its own set of unique interactions between the virus and the cellular membrane system. Members of the tetraspanin (Tspan) protein family are localized to membranes and as such, associate directly and indirectly, with multiple steps of viral infection.

## Tetraspanins

Tetraspanins are a family of small transmembrane proteins (8) that function in cell migration, signal-transduction, intracellular trafficking, and are used by several pathogens for infection (9–11). Of the 33 human Tspans, CD151, CD82, CD81, CD63, CD9, Tspan9, and Tspan7 have been associated with viral infections (12–16).

### Structure

Structurally, Tspans consist of four transmembrane segments, a small extracellular domain, and a large extracellular loop (LEL). Intracellular domains, including the N- and C-terminal tails, are relatively small and contain palmitoylated cysteines. Homology is highly conserved between isoforms with the exception of a small variable domain located within the LEL (17), which may contribute to differences in functionality between isoforms (18, 19).

To date, structural models are only available for CD81. The first model is based on the LEL crystal structure to which the α-helical transmembrane segments were attached in a theoretical conformation. The transmembrane region was predicted to form a four-stranded coiled-coil with two helices extending vertically into the bulkier LEL (20), resulting into a mushroom-shaped structure. The second model, derived from lipidic cubic phase crystallization of the entire protein, describes an arrangement with two major differences. First, instead of assembling into one bundle the transmembrane segments form two coiled-coils resulting in a cholesterol-binding pocket. Second, two kinks exist between the helical transmembrane segments and the LEL, causing the LEL to fold back toward the membrane (21). When cholesterol is released, the kinks straighten, and the LEL adopts an orientation similar to the proposed first model (21).

### Tspan-Enriched Microdomains

Tetraspanins are referred to as master organizers of the plasma membrane, largely due to the fact that they form functional units termed Tspan-enriched microdomains (TEMs or TERMs). Biochemical immunoprecipitation experiments employing detergents of varying strengths revealed two major categories of Tspan interactions: (1) robust interactions between Tspans and non-Tspan binding partners, and (2) weak interactions between Tspan family members (22). Within the second category, certain assemblies of homo–Tspan interactions are preferred over hetero-dimerization/oligomerization (23), and the specificity of oligomerization is mediated by a small segment within the LEL referred to as δ-loop (24, 25). Consistent with these biochemical findings, electron microscopy shows that CD63 and CD9 form distinct clusters (26). Using a more systematic approach, super-resolution light microscopy confirms that single Tspan family members cluster within TEMs (27). Together, these data demonstrate that Tspan isoforms segregate into individual nanoclusters within larger Tspan domains.

In immuno-electron microscopy, Tspan microdomains are highly variable in shape and size with an average surface area of 0.2 µm^2^ (26). When assuming a spherical shape, this corresponds to a diameter of ≈500 nm. In contrast, super-resolution light microscopy detects spherically shaped structures with a diameter in the range of 100–150 nm (27, 28). These two methods result in surface area coverage calculations that differ by more than one order of magnitude. This substantial variability is likely due to the description of multiple nanoclusters within TEMs *via* electron microscopy, whereas super-resolution light microscopy identifies individual nanoclusters due to a higher epitope labeling density.

**Figure 1:**
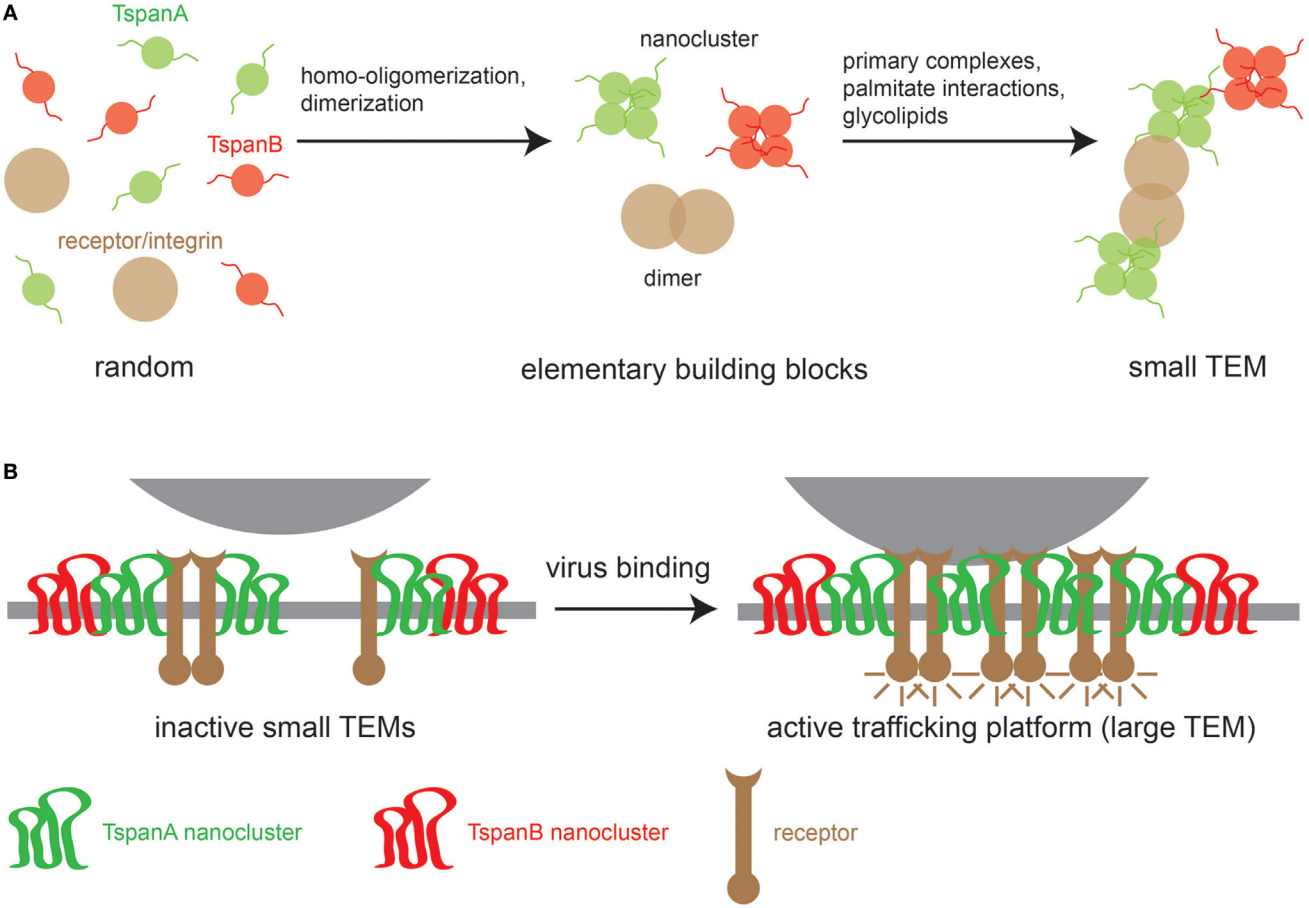
Model of TEM building. **(A)** Left, random distribution of three TEM components: two tetraspanins (TspanA and TspanB) and a primary interaction partner of TspanA [e.g., an integrin or a growth factor receptor (GFR)]. Middle, TEM building blocks; tetraspanins (Tspans) are arranged in homomeric nanoclusters and integrins/GFRs form dimers. Right, building blocks are connected *via* strong specific interactions between TspanA and the receptor/integrin dimer and weak nonspecific interactions between different Tspan nanoclusters. **(B)** When a virus encounters the cellular membrane, virus surface molecules crosslink small TEMs to form larger TEMs, thereby concentrating proteins leading to the activation of intracellular signaling cascades that trigger the uptake of the Tspan trafficking platform.

At present, the sequence of events for TEM biogenesis is unknown, though we can build a model on the following observations. First, different Tspans can associate with each other, but Tspans of one type preferentially homo-oligomerize. Second, Tspans form very tight complexes with non-Tspan partners such as integrins (29, 30) and signaling receptors (31). Finally, TEMs are stabilized by weak nonspecific interactions mediated by the aforementioned palmitate residues (32, 33) and glycolipids that promote Tspan assemblies (24, 34). These different interaction modalities likely produce small TEMs (Figure 1A). Viral surfaces contain abundant identical binding sites that may crosslink small TEMs to large Tspan trafficking platforms (Figure 1B). Evidence for virus-induced large Tspan assemblies has been documented by a number of microscopic studies discussed below.

## Roles of Tspans in Virus Infection

**Figure 2:**
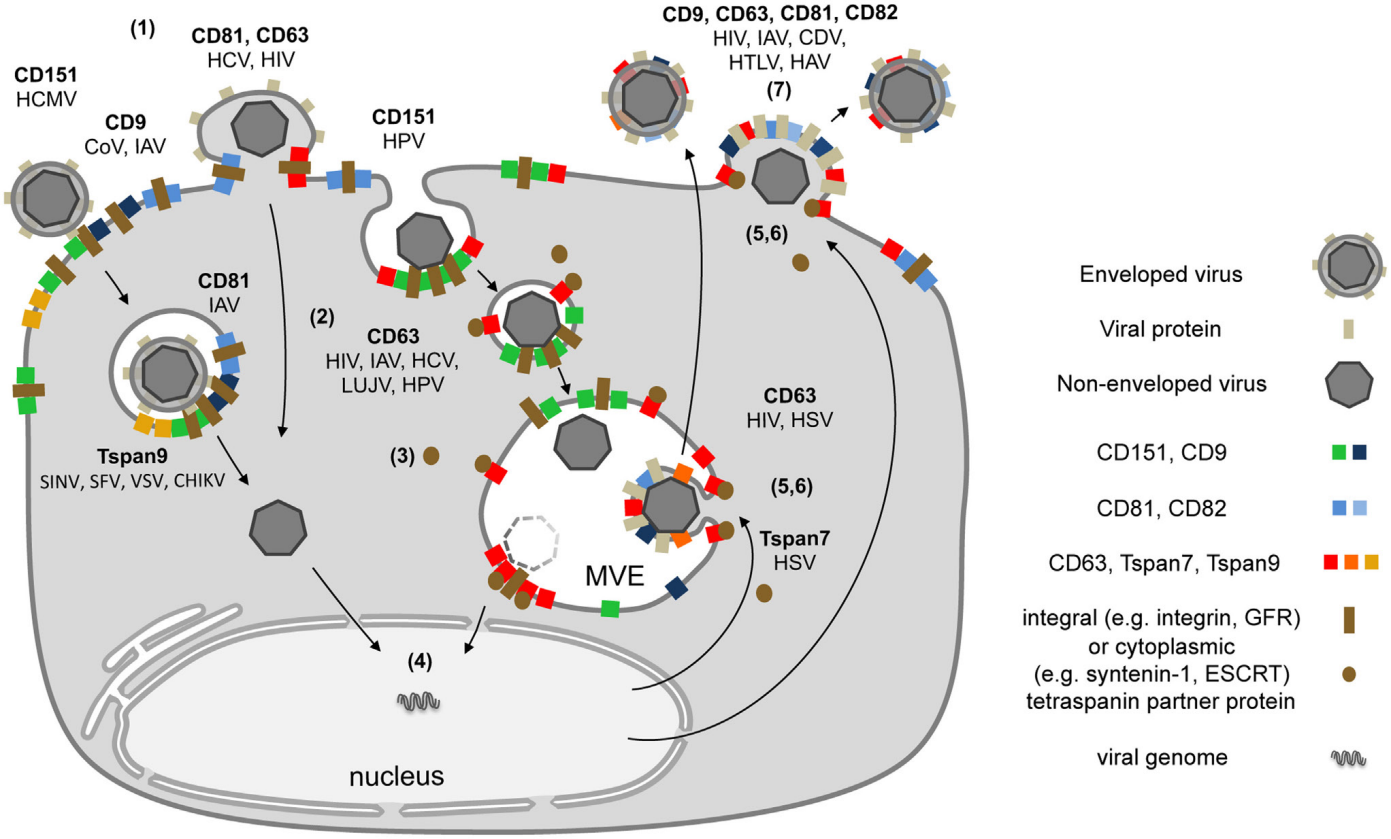
Schematic model depicting the subcellular localization of tetraspanins (Tspans) during virus infection. (1) Interactions between viral particles and entry receptors at Tspan clusters trigger the formation of larger cluster networks. (2) Tspan assemblies promote viral internalization by endocytosis and/or fusion. (3) Endocytosis is followed by intracellular trafficking of virus particles in transport vesicles. During this stage, Tspans mediate and organize interactions with cytoplasmic trafficking molecules. (4) These steps lead to delivery of viral genomes into the cytoplasm or the nucleus and successful infection. (5) Morphogenesis of enveloped viruses on Tspan-enriched microdomains. (6) Integration of viral proteins into Tspan clusters induces spatial enrichment of Tspans and viral proteins. (7) The resulting high concentration of virus envelope components enables efficient budding and release.

Tetraspanins are essential for specific steps in viral entry and exit (12, 13, 15). As described above, contacts between viruses and proteins on the cell surface can lead to large Tspan cluster networks or trafficking platforms (Figure 1B). Similarly, viral envelope proteins accumulate in TEMs during morphogenesis and induce large assemblies of Tspans and viral transmembrane proteins to facilitate efficient budding (Figure 2). These platforms enable the coordination of factors required for viral endocytosis, penetration, trafficking, and release. Here, we summarize and discuss the role of CD151, CD82, CD81, CD63, CD9, Tspan9, and Tspan7 in the life cycle of Tspan-facilitated viruses [for a detailed discussion on the role of Tspans in human immunodeficiency virus (HIV) infection see (Suarez et al.; Tspans, another piece in the HIV-1 replication puzzle) in this issue].

### Tspan Platforms in Virus Endocytosis and Fusion

Studies investigating different viral systems show common mechanisms for how viruses infiltrate their target cells *via* Tspan platforms. Several microscopic studies confirm that Tspans are enriched at viral entry sites of human papillomaviruses (HPVs) (35, 36), hepatitis C virus (HCV) (37–42), coronavirus (CoV), influenza A virus (IAV) (43–45), and HIV (46, 47), and required for penetration of human cytomegalovirus (HCMV) (48) and alphaviruses (16, 49). These viruses use specific Tspans both as receptors and by compartmentalizing host entry factors.

#### CD151 in Early Steps of HPV and Cytomegalovirus Entry

Host cell entry of non-enveloped DNA tumor virus HPV16 relies on a fine interplay between the virion and the host cell. HPV16 binding to primary attachment sites triggers cell signaling events and rearrangement of the viral capsid, membrane proteins, and the actin cytoskeleton (15, 50–52). These processes lead to the formation of a virus entry complex and virus uptake *via* a CD151-dependent and clathrin-independent endocytosis pathway (14, 15, 53). In epithelial cells, surface-bound HPV16 particles colocalize with locally enriched CD151 and CD63 on the plasma membrane during invagination and in endosomes (14, 35). Cellular depletion of CD151 and CD63 leads to significant reduction of infections by different oncogenic HPV types, suggesting that these Tspans play a more general role in HPV entry (35, 36, 54, 55). On T-cells, HPV particles are able to trigger the clustering of CD81 which results in the assembly of larger cluster networks required for particle uptake (56). Furthermore, detailed analyses using CD151 mutants revealed that palmitoylation, the δ-loop of the LEL and the C-teminus of CD151 are critical for HPV16 endocytosis (14, 36). These findings indicate that integration of the virus/receptor-complex into larger TEMs and association with cytoplasmic factors (e.g., actin) are required for this process. HPV endocytosis may also involve interactions between multiple receptors and the viral surface, crosslinking smaller TEMs to larger entry platforms (Figure 1B). HPV16 receptor-complex components include integrins (36, 57–59), growth factor receptors (GFRs) (60), the annexin A2 heterotetramer (61, 62), and other Tspans (35, 36, 56). CD151 directly interacts with integrins and GFRs (14, 22, 63, 64), and, therefore, positions these HPV receptors within TEMs (11, 65). Through this spatial arrangement of functional proteins, CD151 may control enzymatic activities and signaling pathways required for coordinated assembly of the viral entry platform and endocytosis.

Likewise, entry of the enveloped HCMV depends on CD151 and CD151 partner proteins (e.g., integrins, GFRs) and additional Tspans, such as CD9 (15, 48, 66). CD151 is functionally involved post-binding during viral penetration (48). HCMV membrane fusion occurs after clathrin-independent endocytosis in many cell lines (67–69). Together, these studies suggest that CD151-mediated endocytosis might be a prerequisite for efficient HCMV and HPV infection. Mechanistically, virus-receptor, virus-Tspan, and Tspan-Tspan interactions play a vital role in organizing large Tspan platforms, which facilitate coordinated or simultaneous interactions between virus and host to induce membrane invagination by a mechanism yet to be determined [for detailed review see Ref. (15)].

#### CD81 and CD9 in HCV, Corona-, and Influenza-Virus Entry

Similar to HPV and HCMV, HCV entry into hepatocytes is a multistep process involving attachment to cell surface heparan sulfate proteoglycans, conformational changes, and transfer of viral particles to secondary receptors (38, 40, 70, 71). These secondary HCV binding molecules also include integrins, the epidermal GFR (EGFR), the ephrin receptor A2 and Tspans as well as claudin-1 (CLDN1), occludin, the scavenger receptor type B class I (SR-BI), and the serum response factor binding protein 1 (SRFBP1) (72–74). Tspan CD81 plays a multifunctional role in HCV entry. CD81 acts as a virus receptor by directly interacting with the HCV glycoprotein E2 (41, 42). CD81 modulates Tspan interactions after HCV binding (75) by triggering EGFR signaling pathways, which enables Tspan/receptor complex-assembly (76, 77) and promotes CD81-EGFR or CD81-CLDN1 complex formation (74, 76). These events are prerequisite for the endocytosis of CD81-HCV clusters and viral glycoprotein-dependent membrane fusion. Proteomic approaches confirmed complex formation of CLDN1, SR-BI, and SRFBP1 with CD81, and demonstrate the functional requirement of integrin β1 (ITGB1) and SRFBP1 for HCV infection and the physical interaction of the Tspan coreceptor-complex with the signaling molecule HRas (73, 77). The rat sarcoma/mitogen-activated protein kinase signaling pathways and EGFR or EphA2 activity trigger lateral diffusion of CD81 for assembly of the viral entry complex consisting of CD81- CLDN1, HRas, and ITGB1 (74, 77). Because GFRs support the uptake of multiple viruses (78), it is probable that activation of their downstream signaling cascades could trigger Tspan receptor clustering accompanied by cytoskeletal network rearrangement required for the entry of other virus families.

Influenza A-viruses and CoVs are enveloped RNA viruses (79). Tspan microdomains, especially CD81 and CD9 enriched microdomains, are preferred IAV and CoV entry sites as they are required for fusion of viral and host cell membranes in pathogenic infections by both viruses (43–45, 80, 81). IAV and CoV use a variety of coreceptors for this glycoprotein-catalyzed process (82). IAV is routed to CD81-positive endosomes and CD81 is functionally required for the fusion of the viral and the endosomal membrane (45). Here, CD81 may help organize the endosomal membrane and cofactors assisting influenza viral fusion. CoV membrane fusion is mediated by the viral spike glycoprotein (S) and depends on multiple events including proteolytic processing and conformational change of the S protein. Experiments utilizing Tspan knockout cell lines and mice revealed that infection by the human CoV strain 229E requires the Tspan CD9 (43, 44). Pulldown and proximity ligation assays uncovered the four known CoV receptors and a fusion-activating protease within CD9 microdomains. These studies also demonstrated that even in the absence of the virus, CD9 is responsible for the local accumulation of the identified entry receptors. Together, this evidence suggests that CoV uses pre-existing clusters of receptors, proteases, and Tspans for entry. Whether these viruses induce local accumulation of the pre-formed nanoclusters to enable efficient priming of the viral spike proteins during viral egress requires further investigation.

### CD63 and Tspan9 as Regulator of Virus Trafficking and Fusion in Infections by HIV, IAV, HPV, and Lujo Virus (LUJV)

Tetraspanins not only organize plasma membrane molecules but also regulate the trafficking of cellular proteins and the transport of endocytosed viruses (11, 15, 83). Many viruses, including HIV, IAV, HPV, and LUJV, localize to CD63-positive endosomes during entry (35, 46, 55, 81, 84). CD63 is most abundant in late endosomes or multivesicular bodies (MVBs) (85) and involved in the membrane organization and trafficking of cellular transmembrane proteins that interact with viruses such as HIV-receptor component CXCR4 (83, 86, 87). Therefore, a functional involvement of CD63 in viral fusion and transport is conceivable. Cellular depletion of CD63 or treatment of cells with CD63 antibodies leads to decreased infectivity of HIV-1, HCV, LUJV, and oncogenic HPV types presenting CD63 as a more general mediator of virus infection (46, 55, 84, 88–90). In contrast to the proviral role of CD63, it is believed that CD9 and CD81 negatively regulate HIV-1 entry by interfering with the formation of the entry receptor complex (47).

Tetraspanin CD63 forms complexes with HPV16 capsid protein L1 (55). As CD63 is involved in the transport of proteins to multiple subcellular locations, it is plausible that different adaptors are required for regulating its trafficking and sorting. For example, syntenin-1 modulates trafficking of CD63 by binding to its C-terminus (91). Consequently, ultrastructural analyses demonstrated the importance of the CD63/syntenin-1 complex for HPV trafficking to MVBs, a process that is required for capsid disassembly (55). The complexity of CD63-mediated viral trafficking is highlighted by the finding that components of the cellular endosomal sorting complex required for transport (ESCRT) are also integrated into the HPV transport platform (55, 92, 93). ESCRT proteins are able to interact with both, syntenin-1 and viral proteins like the HPV16 capsid protein L2 (55, 92–97). Therefore, both viral and cytoplasmic proteins may be targeted to CD63 platforms in a virus-modulated endosomal trafficking pathway.

In addition to its role in trafficking, CD63 facilitates membrane fusion of enveloped viruses. For example, LUJV glycoprotein-mediated membrane fusion is dependent on CD63 and low pH (84), highlighting the importance of the endo/lysosomal system in cell entry. Similarly, Tspan TSPAN9 promotes membrane penetration in early endosomes by the alphaviruses Sindbis virus, Semliki Forest virus, vesicular stomatitis virus, and chikungunya virus (16, 49). Together, CD63 and TSPAN9 may modulate the endosomal compartment to be more permissive for the fusion of viral and cellular membranes.

### Tspans in Virus Exit

Morphogenesis of enveloped viruses occurs on membranes of intracellular compartments or at the plasma membrane. Like virus entry, virus exit is a multi-step process driven by viral proteins. This process includes the targeting of viral proteins to specific membrane domains, local concentration of these proteins, virus budding, and release of virus particles. During these processes, Tspans are incorporated into the enveloping membrane of virions, such as HIV, feline immunodeficiency virus, canine distemper virus (CDV), HCMV, influenza, or hepatitis A virus (HAV) (98), implicating TEMs at the site of virus budding.

Earlier reports support this hypothesis using electron and fluorescence microscopy to demonstrate that the HIV core (Gag) and envelope (Env) proteins (26, 99–101), the HTLV-1 Gag protein (102, 103), the Marburgvirus matrix protein VP40 (104, 105), and influenza proteins (45) accumulate in CD9, CD63, CD81, and/or CD82 containing TEMs.

Studies investigating Tspan dynamics in virus budding have shown that the herpes simplex virus (HSV)-1 capsid protein VP26 physically interacts with Tspan7 (earlier known as CTMP-7) (106), and that formation of this complex supports viral egress. Moreover, influenza infection induced redistribution of CD81 on the plasma membrane into concentrated patches of viral budding sites which also contain different viral proteins (45). Likewise, CD63 coordinates sorting of specific viral proteins into extracellular vesicles, such as the major oncoprotein latent membrane protein 1 of the Epstein–Barr virus (107, 108). Comparable to influenza budding, HIV Gag insertion into the plasma membrane induces recruitment of CD81 and CD9 and the coalescence of different membrane microdomains (100, 101, 109–111). Co-immunoprecipitation experiments revealed that Gag proteins interact, directly or indirectly, with CD81 (100). The process of Gag accumulation in Tspan assemblies leads to the formation of larger membrane domains that extend over a few hundred nanometers (109, 112) and contain up to 2,500 tightly packed Gag molecules (113). In addition, multiple studies have shown that modulation of Tspan expression levels and redistribution *via* anti-Tspan antibody treatment in viral or cellular membranes interferes with different steps of the HIV and CDV life cycle including virus-to-cell fusion, reverse transcription, release, and virus-induced cell–cell fusion (114–121). Thus, Tspans can regulate, for example, viral release and cell–cell fusion by controlling the access of the required cellular machineries to the specific areas.

In addition to Tspans and viral proteins, HIV and HAV exit platforms accumulate cytoplasmic factors, such as components of the ESCRT machinery, which are required for the budding process (113, 122–127). This Tspan-mediated pre-assembly of viral and cellular proteins enables the formation of large budding platforms, a precondition for coordinated viral morphogenesis.

## Conclusion

At present, the various interaction modalities between viral and cellular proteins preclude the development of a simple model for viral entry. Common molecular mechanisms in viral infection may be revealed by characterizing Tspan platforms in different systems, from their initial involvement at the plasma membrane to their roles in intracellular trafficking and viral egress (Figure 2). We hypothesize that active accumulation of molecules into Tspan platforms drives viral infection forward in a defined step-wise sequence.

## Author Contributions

LF and TL wrote the manuscript and designed the figures.

## Conflict of Interest Statement

The authors declare that the research was conducted in the absence of any commercial or financial relationships that could be construed as a potential conflict of interest.

## Abbreviations

CD, cluster of differentiation; CDV, canine distemper virus; CLDN1, claudin; CoV, coronavirus; EBV, Epstein–Barr virus; ESCRT, endosomal sorting complexes required for transport; FIV, feline immunodeficiency virus, GFRs, growth factor receptors; HAV, hepatitis A virus; HCMV, human cytomegalovirus; HCV, hepatitis C virus; HIV, human immunodeficiency virus; HPV, human papillomavirus; HSV, herpes simplex virus; IAV, influenza A virus; ITGB1, integrin β1; LEL, large extracellular loop; LUJV, Lujo virus; MAPK, mitogen-activated protein kinase; MVB, multivesicular body; Ras, rat sarcoma; SR-BI, scavenger receptor type B class I; TEMs or TERMs, tetraspanin-enriched microdomains; TIRF, total internal reflection fluorescence microscopy; TM, trans-membrane; Tspan, tetraspanin; vDNA, viral DNA.
